# Theoretical framework for soft X-ray Fourier transform spectroscopy using the Wigner function

**DOI:** 10.1107/S1600577525011452

**Published:** 2026-01-30

**Authors:** Chuzida Chen, Andrew Lindburg, Honghe Ding, Antoine Wojdyla, Howard Padmore, Per-Anders Glans, Jinghua Guo

**Affiliations:** a Advanced Light Source Lawrence Berkeley National Laboratory 1 Cyclotron Road Berkeley CA94720 USA; b University of California Berkeley CA USA; c Physics Department Arizona State University Tempe AZ85282 USA; Tohoku University, Japan

**Keywords:** Fourier transform spectroscopy, soft X-rays, theoretical demonstrations of Fourier transform spectroscopy

## Abstract

This work presents a theoretical framework for the propagation of partially coherent Gaussian radiation in a modified Mach–Zehnder interferometer designed for Fourier transform spectroscopy (FTS) applications. Using the Wigner function formalism, we analytically propagate the radiation through the system and benchmark our approach by comparing the resulting interference pattern and interferogram with previous works in the diffraction limit. Our analysis reveals that the transverse coherence length requirement of the incident light field for detectable modulation is less stringent than previously assumed. Additionally, we provide theoretical demonstrations of FTS performance across various wavelengths using the proposed setup. These findings underscore the potential of this interferometer to achieve high-resolution FTS in the soft X-ray regime.

## Abstract 2

A theoretical framework for the propagation of partially coherent Gaussian radiation in a modified Mach–Zehnder interferometer designed for Fourier transform spectroscopy applications is presented.

## 1. Introduction

The technique of Fourier transform spectroscopy (FTS) has been widely used in the infrared region and even in the optical region, primarily because high resolution can be achieved in a facile way by introducing a scanned path length change into one of the arms of an interferometer. Spectra are then recovered from the Fourier transform of the intensity as a function of the path length change, and the resolving power is only limited by the ratio of the maximum path length difference to the wavelength. The fundamental component that enables FTS to operate in the long-wavelength regime is an amplitude-dividing beam splitter, typically a partially transmitting and reflecting metal-coated glass surface. To extend FTS into the vacuum ultraviolet (VUV) and soft X-ray (SXR) regimes, this approach cannot be used because of absorption in the thin metal film and the beam-splitter substrate material. In the early 1990s, Howells *et al.* (1994 ▸) proposed and built a system for VUV FTS at 65 eV using a modified Mach–Zehnder arrangement to study the double ionization series of helium (Moler *et al.*, 1997 ▸). The conventional beam splitter used at long wavelengths was replaced by a very coarse transmission-grating-shaped structure, allowing light to be bifurcated through wavefront division. Due to mechanical limitations that involve extreme fabrication tolerances, alignment tolerances, and overall complexity, this system did not achieve its theoretical performance. However, in 2010, a group at Synchrotron Soleil achieved a resolving power of 1 × 10^6^, using a much simpler wavefront dividing interferometer (Oliveira *et al.*, 2011 ▸). This was based on a Fresnel bi-prism, in which one of the 180° roof mirror elements was scanned in position relative to the other, and the tilt of one roof mirror was used to recombine the scanned and unscanned beams in the far field, obviating the need for a conventional beam splitter. Due to the use of two 45° reflections to create the overall 180° deflection, this design was only suitable up to around 40 eV, due to the reflectivity cutoff of gold. In addition, this system employed pure wavefront division, with the upper and lower halves of the roof mirrors each receiving 50% of the beam and requiring coherent illumination.

In this work, we have revisited the original Mach–Zehnder style of FTS. The reason for this is that it was never completely clear how coherent the beam had to be and how the beam-splitting and beam-recombining gratings worked in tandem to produce the desired interference at the detector. In further analysis, the system is a hybrid of wavefront and amplitude-dividing systems, and therefore the requirement for coherent illumination is substantially relaxed. This opens the possibility for application in resonant inelastic X-ray scattering (RIXS) (Agåker *et al.*, 2011 ▸) where a resolution beyond that achievable with grating spectrographs is desirable. We have therefore examined a system similar to the original Howells scheme and simulated the transmission of partially coherent light to investigate how fringe modulation depends on the degree of coherence. Previous studies by Yin *et al.* (2000 ▸), based on classical grating diffraction theory with the assumption of coherent illumination, showed that the coherence width of the beam should be at least equal to two periods of the grating beam splitter. We have built upon this work by using the Wigner function formalism while rigorously incorporating coherent illumination. Specifically, our findings suggest that the coherence constraints may be less stringent than previously estimated. The initial section reviews the formalism, while the rest of the paper derives the expected interference pattern and interferogram. Finally, we motivate the study by demonstrating the projected performance of the device at soft X-ray wavelengths through the interferogram.

## 2. Wigner function formalism

The application of the Wigner function formalism in analyzing the propagation properties of synchrotron radiation was first introduced by K. J. Kim (Kim, 1986*a* ▸), where it was referred to as ‘brightness’. Similar to its origin in quantum optics (QO) (Wigner, 1932 ▸), the Wigner function for electromagnetic radiation is a quasi-probabilistic phase-space distribution that has been proven effective in modeling the propagation of partially coherent radiation; for a detailed comparison between QO and the classical light-field Wigner function see Bazarov (2012 ▸).

Our work requires an understanding of both interference effects and coherence properties to derive the interferogram accurately. While the van Cittert–Zernicke theorem (VCZT) is conventionally used to handle the coherence properties of propagating radiation, the Wigner function formalism offers a distinct advantage by embedding both interference and coherence information within a unified framework.

It is also worth noting that the interferometer configuration is close to the Fresnel regime in which diffraction is not yet fully developed. This makes the Wigner function formalism a suitable model for analyzing the system. This aspect will also become increasingly relevant as beamlines become more coherent, thanks to the worldwide upgrade of synchrotron facilities.

The Wigner distribution function (WDF) for fully coherent EM radiation having an electric field *E*(*x*) and wavelength λ is defined as (Nash *et al.*, 2021 ▸)
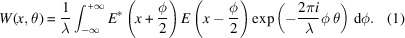
This expression corresponds to applying the Wigner–Weyl transform, denoted as 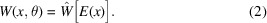
For a partially coherent field, the WDF generalizes to include statistical fluctuations,
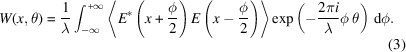
In 2D, 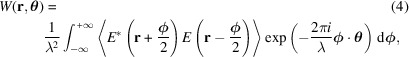
where 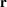 = 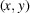 is the transverse coordinate and 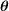 = 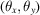 is the angle of deviation from the optical axis; 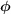 is also a 2D vector with dimension of length. The brackets denote the ensemble average, and the function should be normalized to 1 upon integration throughout the phase space (Kim, 1986*a* ▸).

### 2.1. Transformation properties of the Wigner function

Similar to the propagation of the light field, its associated Wigner function also propagates along a beamline. There are two cases to be considered: free space propagation and metamorphism upon the encounter of an aperture. The derivation in this section will be done in 1D without a loss of generality.

#### 2.1.1. Free space transformation

Beginning with free space propagation, the Fresnel diffraction formula governs the free space propagation of the electric field in position space (Born *et al.*, 1999 ▸). To simplify the derivation, we introduce the Fourier transform of the electric field in the angular domain, 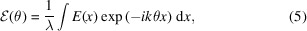
where 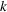 = 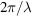. Under this definition, equation (3) would become 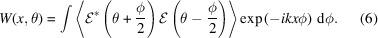
Assuming that the field propagates with small angular divergence for a distance *l*, the equivalent free space propagation equation for the angular domain is (Kim, 1986*a* ▸)
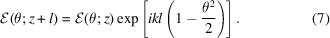
Note that *z* is the longitudinal position. The subsequent Wigner function is as follows, 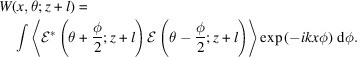
Substituting equation (7) into the above equation and simplifying, we find 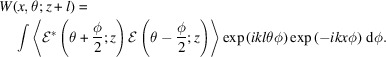
Finally, applying the Fourier transform property that, for a Fourier conjugate pair *x* and θ, if 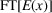 = 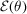 then 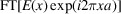 = 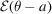, one arrives at the evolution formula for the Wigner function upon paraxial propagation in free space (Kim, 1986*a* ▸), 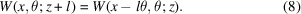
In another paper, K. J. Kim highlights that the free-space propagation rule of the Wigner function is the equivalent of VCZT (Kim, 1986*b* ▸). See the supporting information for a detailed derivation of the above statement and how one can derive an extended version of the Generalized van Cittert–Zernike theorem (GVCZT) using the Wigner function.

Graphically, equation (8) amounts to a shear in the phase space. This provides a clear intuition of how the optical path difference introduced by the device manifests within the Wigner function formalism. Specifically, as will be seen later in Figs. 3 and 4, the propagated WDF exhibits characteristics that resemble two differently sheared Wigner functions superposed on each other. The relative shear between these contributions encodes the optical path difference (OPD), and it is precisely this difference that underlies the emergence of interference.

#### 2.1.2. Encountering an aperture

The derivation for transforming the Wigner function upon encountering a physical aperture is similar. The resultant electric field after the aperture would be the original field multiplied by the transfer function (or the shape) of the aperture,
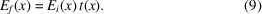
Therefore, substituting the above expression into equation (3), the subsequent WDF is 
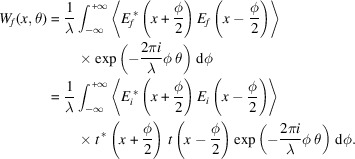
Note that, from the definition of the Wigner function, we know that 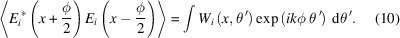
Using this, *W*_*f*_(*x*, θ) can be rearranged as follows,
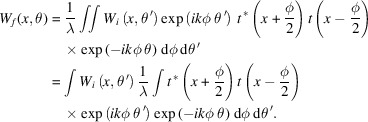
Again, using the Fourier transform property in the derivation of the free-space evolution of the WDF, we find that, upon encountering an aperture of the transfer function *t*(*x*), the updated WDF is
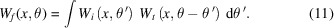
This is essentially a partial convolution over the angular component between the original Wigner function and 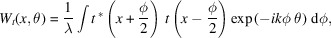
which is the Wigner–Weyl transform of the aperture transfer function (Bazarov, 2012 ▸). *W*_*t*_(*x*, θ) is called the aperture Wigner function or the Wigner filter function (Nash *et al.*, 2021 ▸).

### 2.2. The cross Wigner–Weyl transform

Before continuing, it is essential to introduce an important concept that will be discussed later: the cross Wigner–Weyl transform, also known as the cross Wigner function. The cross Wigner function originates from the non-linearity of the Wigner–Weyl transform. Suppose that the total field *E*_tot_ is the superposition of two fields *E*_1_ and *E*_2_. Then the Wigner–Weyl transform of *E*_tot_ is given by equation (4),
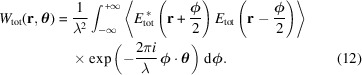
Inserting 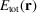 = 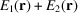 into the above equation and simplifying yields the following relation between the total Wigner function and individual Wigner–Weyl transforms of field *E*_1_ and *E*_2_,
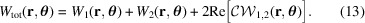
The above expression indicates that the combined Wigner function associated with the superposition of fields 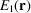 and 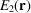 would be the addition of the Wigner function they are respectively associated with—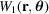 and 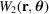
—along with the cross Wigner function of *E*_1_ and *E*_2_. The cross Wigner function 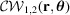 is defined as 
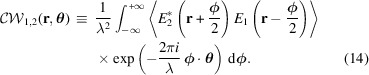
As one may observe, the cross Wigner function is itself the interference term in the formalism, and it captures interference effects between the two fields. In the case when *E*_2_ is essentially *E*_1_ with a complex phase, the cross term can be expressed solely in terms of *W*_1_ with an additional phase factor.

### 2.3. Interference patterns

As a more general interference law highlighted by Agarwal (1995 ▸), the Wigner function captures the interference pattern generated by the incident radiation in both position and angular space. This can be directly derived from equation (3) by projecting the Wigner function on either phase space variable.

For example, using the identity 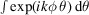 = 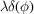,
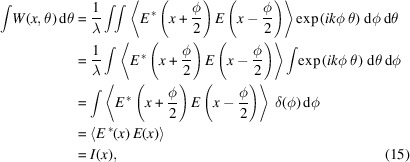
with *I*(*x*) being the intensity distribution. Using the same approach, one can show that (Bazarov, 2012 ▸)
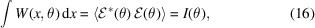
for *I*(θ) being the far-field angular intensity distribution (Bazarov, 2012 ▸). Therefore, to find the interference pattern due to some partially coherent radiation, one can derive the original Wigner function at the source point, propagate the WDF along the beamline, and finally project the WDF on either the position variable or the angular variable to derive the interference pattern on the imaging plane.

As demonstrated, knowledge of the Wigner function can lead to insights into both the interference pattern and the spatial coherence function. Calculations associated with this formalism will therefore be of the following algorithm:

(i) Derive the Wigner function at the source.

(ii) Propagate the Wigner function through the beamline/optical system.

(iii) Integrate over the angular or position variables for the interference pattern or perform an inverse Fourier transform for the mutual coherence function.

## 3. The Mach–Zehnder type interferometer

**Figure 1:**
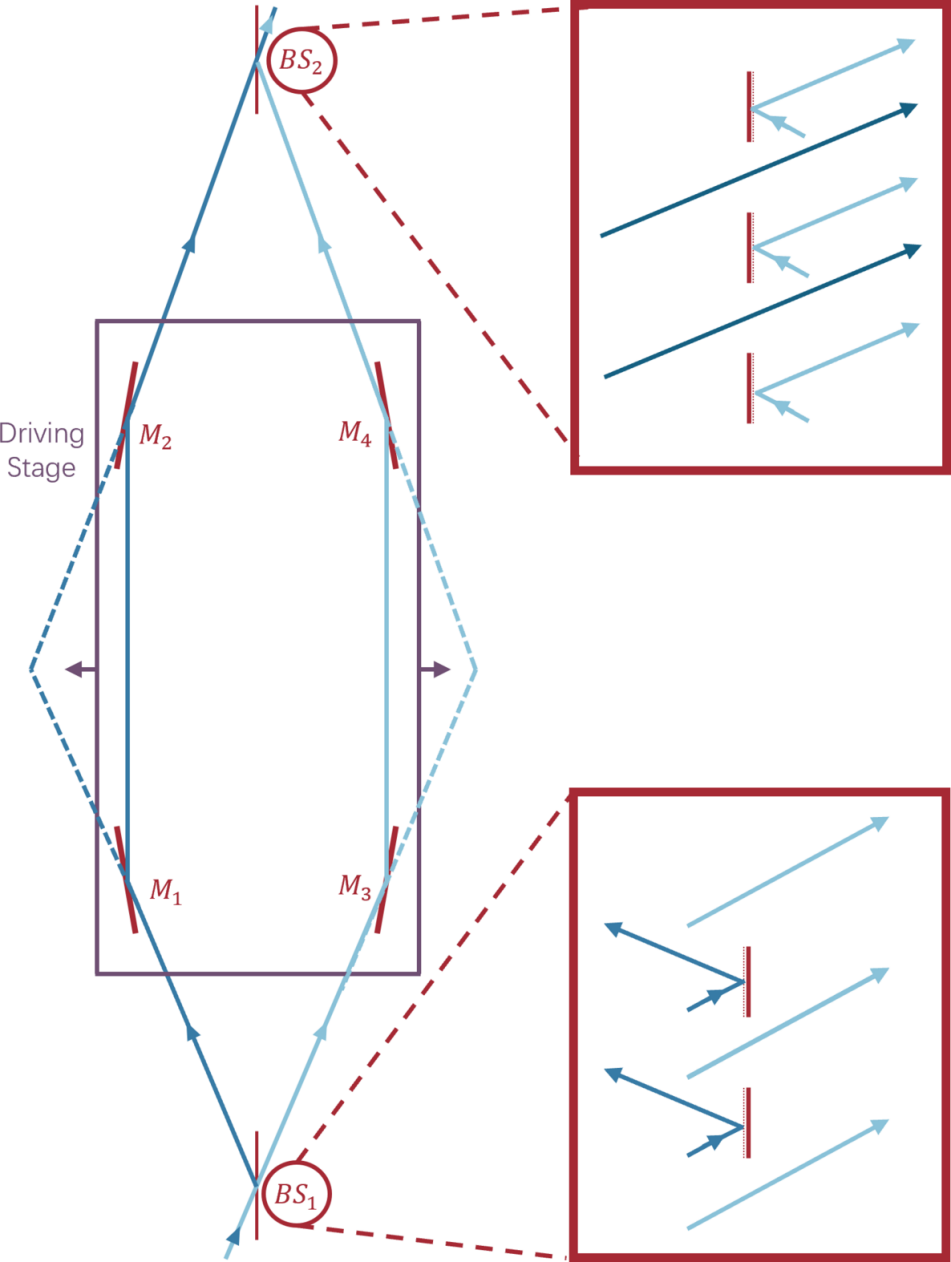
Left: scheme of the soft X-ray FTS device. *BS*_1_ and *BS*_2_ are beam splitters of the same structure with a one-grating-period offset relative to each other. *M*_1_, *M*_2_, *M*_3_, and *M*_4_ are mirrors that form the rhombus geometry. The beam path is highlighted in blue, and the driving stage is highlighted in purple. Top right: scheme of the second beam splitter *BS*_2_ (the beam combiner). Light blue is light from the *M*_3_*M*_4_ arm that is reflected at *BS*_2_, and dark blue is light from the *M*_1_*M*_2_ arm transmitting through. Bottom right: scheme of the first beam splitter *BS*_1_. Light blue represents light from the source being reflected into the *M*_1_*M*_2_ arm, and dark blue is radiation transmitting through, into the *M*_3_*M*_4_ arm of the FTS device.

A schematic of the setup under analysis is shown on the left in Fig. 1 ▸. The dashed lines extending along the beam path show the rhombic geometry. The advantage of such an arrangement lies in its ability to introduce a long OPD between the bifurcated beams upon horizontal translation of the flexure stage, relative to the beam splitters (Wilcox *et al.*, 2010 ▸); this allows the interferometer to reach a high resolving power coupled with a high degree of control.

The beam splitters, labeled *BS*_1_ and *BS*_2_, and the flat mirrors *M*_1_, *M*_2_, *M*_3_, and *M*_4_ are arranged to produce grazing-incidence reflections. In the original setup by Howells *et al.*, they employed transmission-grating-shaped beam splitters, featuring reflective bars and micro-fabricated transmissive slots, created by etching a single silicon crystal with an alkali solvent (Howells *et al.*, 1994 ▸). The beam splitter schemes are shown in the upper right (for *BS*_2_) and lower right (for *BS*_1_) portions of Fig. 1 ▸, similar to what was presented by Agåker *et al.* (2009 ▸). In the current configuration, *BS*_1_ and *BS*_2_ are composed of thin films of silicon nitride perforated with small, densely packed holes, which allow for the minimization of stress within the material, providing sufficient flatness for the coherence of the recombined beam (Wilcox *et al.*, 2010 ▸).

## 4. The aperture Wigner function

Before applying the formalism to propagate Gaussian radiation, we first construct the Wigner aperture function for the beam splitters. Based on the given scheme (Fig. 1 ▸), the beam splitter (BS) can be mathematically represented as a superposition of rectangular functions at various positions. The rectangular function is defined as 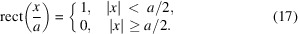
The plot of this function produces a rectangular peak of width *a*, centered on *x* = 0. Using rect(*x*), the aperture function of *N* even slits, separated by a distance *b*, is expressed as
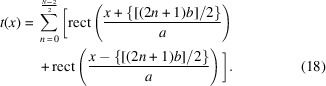
The corresponding *W*_*t*_(*x*, θ) can then be derived by applying the Wigner–Weyl transform to this function. The solution for the arbitrary case can be illuminated by considering the following two integrals: the Wigner–Weyl transform of single slits and the cross Wigner function between 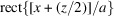 and 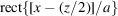.

**Figure 2:**
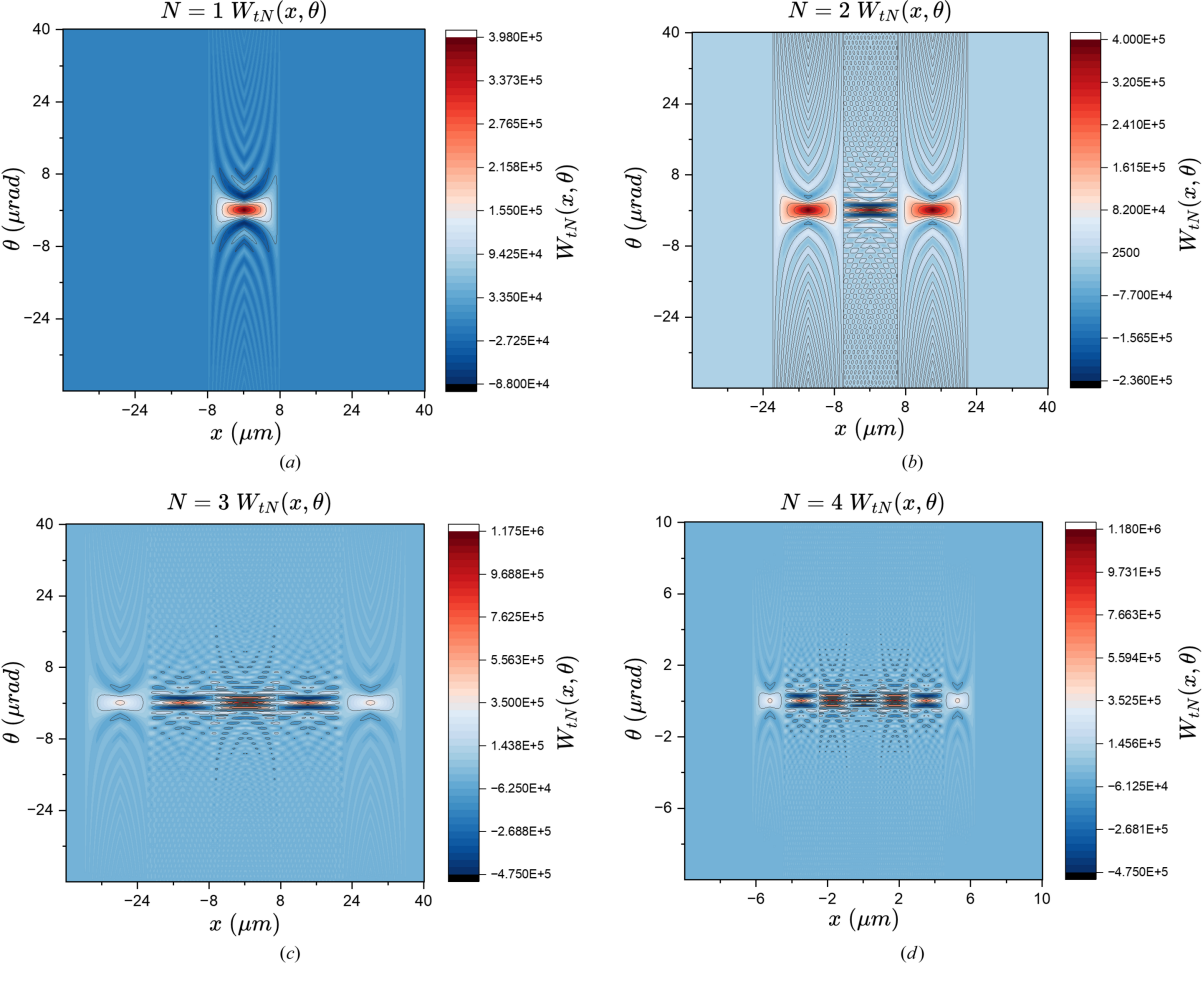
Aperture Wigner functions of various *N* values. Other parameters are 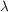 = 1 Å, *a* = 20 µm, *b* = 35 µm. (*a*) *N* = 1, (*b*) *N* = 2, (*c*) *N* = 3, (*d*) *N* = 4.

The Wigner–Weyl transform integral of the single-slit aperture function is 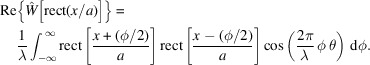
The real part is taken because we expect real results. Observe that 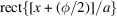 is nonzero when ϕ is in the range −*a* − 2*x* < ϕ < *a* − 2*x*;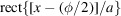 is nonzero only if −*a* + 2*x* < ϕ < *a* + 2*x*. Therefore, the integral above can be condensed into 
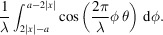
Finally, the integral evaluates to the following solution, 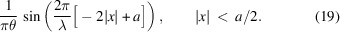
This result can be generalized to any shifted slits, say 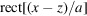; the Wigner function will be of the following form, 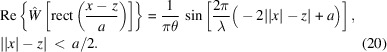
The cross Wigner–Weyl transform between 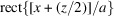 and 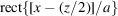 is defined as
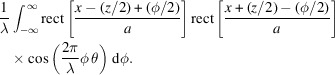
Note that rect{[*x* − (*z*/2) + (φ/2)]/*a*}rect{[*x* + (*z*/2) − (φ/2)]/*a*} is nonzero if and only if −*a* + 2|*x*| + *z* < ϕ < *a* − 2|*x*| + *z*. Therefore, we have 
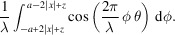
This integral yields the result
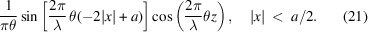
This expression can be further metamorphosed into a more illuminating form. If we replace *x* with 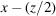, then we derive the cross Wigner function between two rectangle functions, one at the center and one shifted,
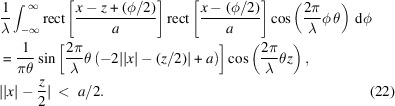
Finally, using the same approach, we arrive at the cross Wigner function between any two rectangle functions at arbitrary positions of *z* and −*y* (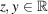),
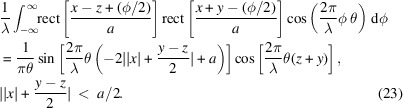
Equations (20) and (23) are going to help us compute *W*_*t*_(*x*, θ), associated with *N* even slits. The steps are as follows,
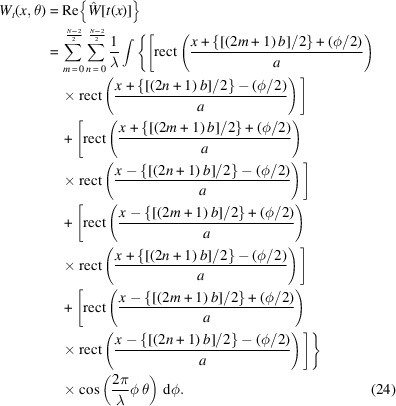
The double summation originates from evaluating equation (18) in the form of 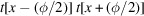 for the Wigner–Weyl transform. *m* is assigned to label the summed rectangular functions in 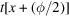 while *n* labels what belongs to 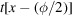. Now we can invoke equation (23) in each of the four terms of the integral. With some simplification, we find the following,
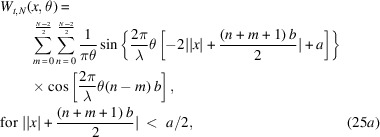

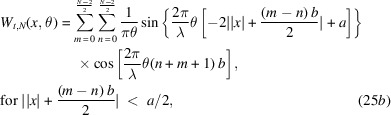

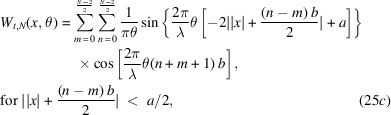

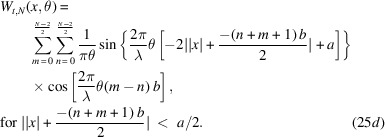
The aperture Wigner function for arbitrary *N* odd slits can be derived similarly. The aperture function for *N* odd slits is
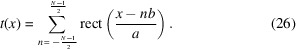
Performing the Wigner–Weyl transform on equation (26) and simplifying using equation (23) yields 
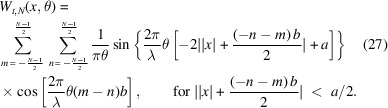
This result is plotted in Fig. 2 ▸ assuming that 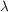 = 1 Å, *a* = 20 µm, *b* = 35 µm, and various values of *N*.

Equations (25) and (27) are the main results of this section. They provide the aperture Wigner functions for even and odd *N* slits, respectively. These equations extend and generalize the aperture Wigner functions discussed by Nash *et al.* (2021 ▸) and Román-Moreno *et al.* (2003 ▸).

## 5. Propagating a partially coherent radiation

### 5.1. Method of analysis

In our approach, a 2D phase space is assumed, as only the direction along the grooves is relevant. One can assume a 4D phase space for an even more exhaustive simulation. Suppose that the source radiation is characterized by the Wigner function 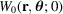. At *BS*_1_, located a distance *z*_1_ from the source, the incident WDF is obtained via equation (8). For clarity, the coordinate *z* resets to zero after each BS. Given that *BS*_1_ is assigned to perform intensity division (Yin *et al.*, 2000 ▸), using equation (4), the Wigner function associated with radiation reflected into the *M*_1_*M*_2_ arm would be 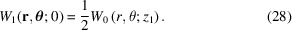
Similarly, radiation transmitted into the *M*_3_*M*_4_ arm is associated with the following WDF, 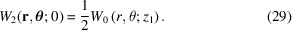
The two Wigner functions *W*_1_ and *W*_2_ then propagate in free space for a distance of, respectively, *z*_2_ (the *M*_1_*M*_2_ arm) and *z*_3_ = *z*_2_ + *d* (the *M*_3_*M*_4_ arm). *d* is the OPD that gives rise to the phase difference, ϕ = *kd*, between light from the two arms, leading to an interference pattern; *k* is the wavenumber of the source field. At the second BS we would have 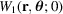

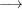

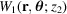
 and 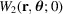

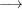

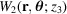
.

The transfer function 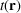 of *BS*_2_ first modifies *W*_1_ into 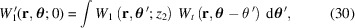
with *W*_*t*_ being 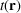
’s Wigner–Weyl transform. The reflection of beams from the *M*_3_*M*_4_ arm is also modeled by equation (11). In this case, the convolving kernel is taken to be the Wigner–Weyl transform of the transfer function shifted by a period/slit width along the transverse direction *W*_*t*,*a*_. Therefore, *W*_2_ becomes
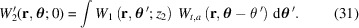
After *BS*_2_, the combined WDF is given by the sum of 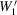 and 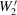, together with an interference term originating from the cross Wigner–Weyl transform of the two light fields, which incorporates their relative phase difference. Therefore, the final WDF is 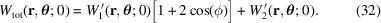
Subsequently, the propagation law can be used to evolve the combined Wigner function to the detector a distance *z*_4_ away. The far-field angular interference pattern is then the θ-projection of 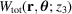. This scheme of beam division and recombination is consistent with that presented by Yin *et al.* (2000 ▸). As we shall see, this consistency is the key to the agreement between the two approaches in the coherent limit.

### 5.2. Partially coherent Gaussian radiation

The Wigner function associated with a partially coherent Gaussian radiation is of the following form, 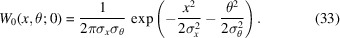
The position divergence σ_*x*_ and the angular spread σ_θ_ obey the diffraction limit formula, 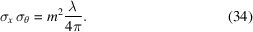
This is the classical correspondence of the quantum-mechanical uncertainty relation. The parameter *m*^2^ is called the beam quality factor (Nash *et al.*, 2021 ▸); in the case of *m* > 1, the light is partially coherent, and, if *m* = 1, it is diffraction-limited (coherent).

The Gaussian then propagates a distance before encountering *BS*_2_. According to the proposed procedure, the positionally shifted Gaussian Wigner function will be angularly convoluted with a Wigner function of an *N*-slit aperture. A useful simplification for mathematics is shown below, 
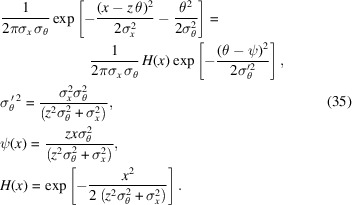
Originally, as covered by Nash *et al.* (2021 ▸) (see their Appendix *E*), the angular convolution between a Gaussian and a scaled sinc function is 
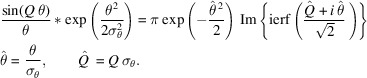
Note that *Q* is a constant from the perspective of θ and, in our case, could depend on *x*. Using the shift property of convolution, the result above can be easily applied to a shifted Gaussian, 
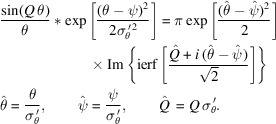
Since all aperture Wigner functions (both even and odd) are of the form 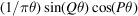, using the equation above, it is convenient to have the identity that
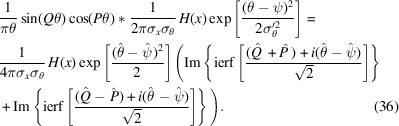
Using equation (36), the analytical expression of the propagated Wigner function is shown in Appendix *B* of the supporting information.

### 5.3. Connecting *m*^2^ to the coherence width

From the definition of the Wigner function, one can see that the spatial coherence function (the two-point correlation of) a light field is related to its associated Wigner function by
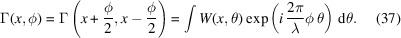
Since our WDF is associated with a Gaussian radiation function of the form 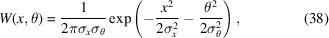
inserting this Gaussian into the above equation, solving and replacing *x* and ϕ with 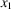 = 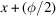 and 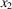 = 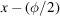 yields
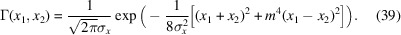
According to the definition of the spatial coherence function of a partially coherent light field (Goodman, 2000 ▸),
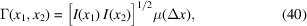
in which μ(Δ*x*) is the complex degree of coherence. One can obtain the expression of μ(Δ*x*). Since we know
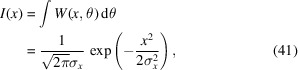
we can find that
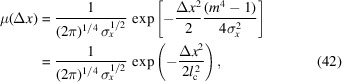
in which the transverse coherence length is given by 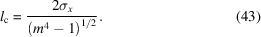
This is precisely the classical analog of what was derived by Bazarov (2012 ▸) for a pure Gaussian ground state. It is important to note that this *l*_c_ is not the traditional transverse coherence length, in which any evaluation of the two-point correlation with Δ*x* beyond *l*_c_ would yield zero. Instead, due to the Gaussian form of the complex degree of coherence, instead of a Dirac delta, the correlation for any pair of points with Δ*x* > *l*_c_ is not necessarily 0; it decreases following a Gaussian as Δ*x* increases.

### 5.4. Results

**Figure 3:**
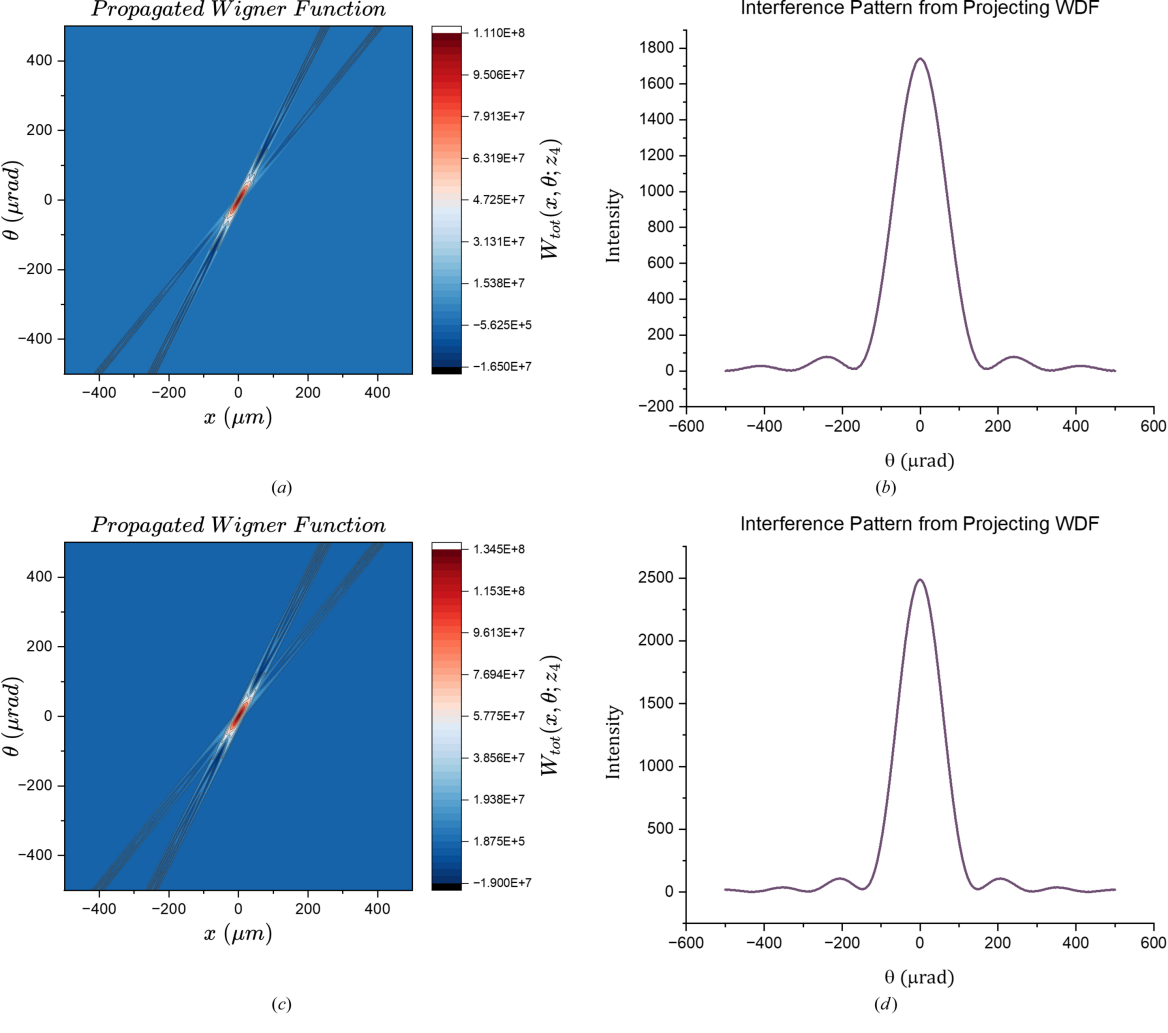
Shown in the left column are the propagated Wigner functions; on the right are the respective interference pattern predicted by such WDF. Parameters for (*a*) and (*b*): λ = 50 Å, *m*^2^ = 3, *N* = 3, *a* = 10 µm. Parameters for (*c*) and (*d*): λ = 50 Å, *m*^2^ = 3, *N* = 5, *a* = 7 µm.

Fig. 3 ▸ shows examples of propagated Wigner functions and the far-field angular interference pattern determined by projecting the WDF onto the θ variable. The simulation parameters are connected to their respective physical quantities in Table 1 ▸.

**Figure 4:**
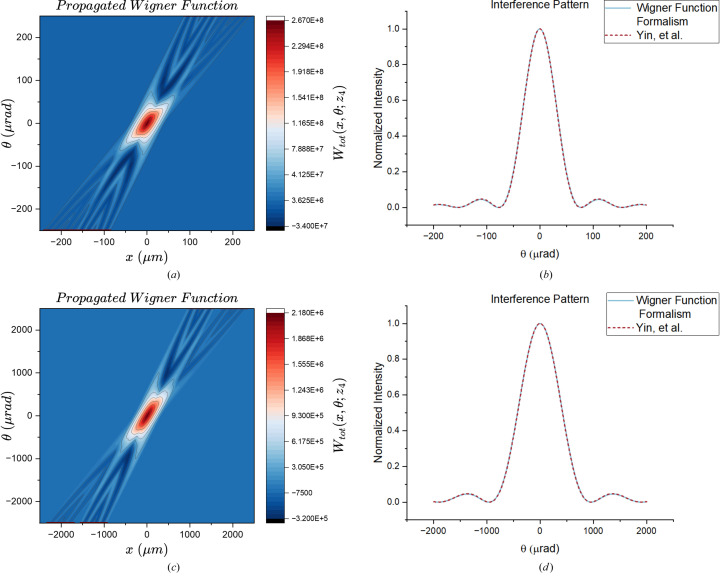
Comparison of the propagated, coherent WDF (on the left) projections versus the interference pattern derived in Yin *et al.* (2000 ▸). The two curves agree with each other. The drift lengths for both cases are fixed: *z*_1_ = 10 cm, *z*_2_ = 30 cm, *z*_4_ = 0.5 m, along with OPD = 1 cm. Parameters for (*a*) and (*b*): λ = 50 Å, *m*^2^ = 1, σ_*x*_ = 75 µm, *a* = 13 µm, *N* = 5. Parameters for (*c*) and (*d*): λ = 5000 Å, *m*^2^ = 1, σ_*x*_ = 750 µm, *a* = 75 µm, *N* = 7.

In the case of *m*^2^ = 1, equation (33) corresponds to coherent radiation. Therefore, comparing the far-field interference pattern predicted by the Wigner function formalism in this limit with the results presented by Yin *et al.* (2000 ▸) serves as a valuable verification. This comparison focuses on the shape of the peaks, as the two formalisms use different normalizations. If the full width at half-maximum (FWHM) and the position of the peaks are identical, the peaks can be considered equivalent. Fig. 4 ▸ illustrates comparisons between the projections of the propagated Wigner function and the interference pattern generated by coherent sources.

Graphs within Fig. 4 ▸ demonstrate that the FWHM of the primary intensity peak predicted by the Wigner function matches that of the coherent case. Not only that, but the two interference patterns are in excellent agreement with each other.

**Figure 5:**
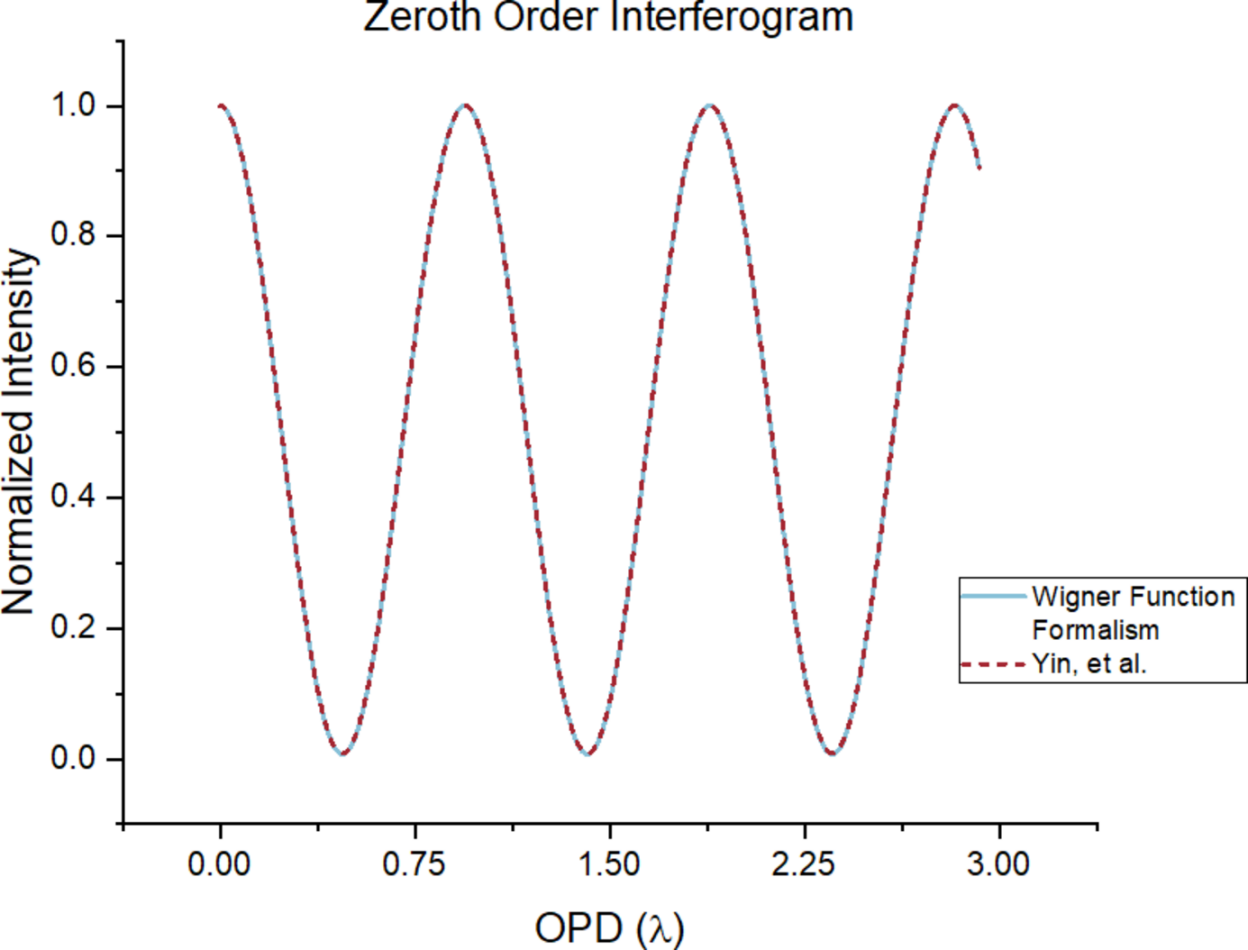
Interferogram comparison between the WDF formalism and equations from Yin *et al.* (2000 ▸). Simulation parameters: *z*_1_ = 10 cm, *z*_2_ = 30 cm, *z*_4_ = 0.5 m, σ_*x*_ = 75 µm, λ = 50 nm, *a* = 6 µm, *N* = 11, *m*^2^ = 1. As shown by the figures, the two formalisms agree with each other.

As a further verification, shown in Fig. 5 ▸ is a comparison of the interferogram predicted by our formalism in the limit of *m*^2^ = 1 along with that predicted by the equations of Yin *et al.* (2000 ▸). The analysis region is focused on the zeroth-order peak, and the OPD is sampled in the range of 0 to 3 times the wavelength. The two formalisms indeed predict the same modulation.

**Figure 6:**
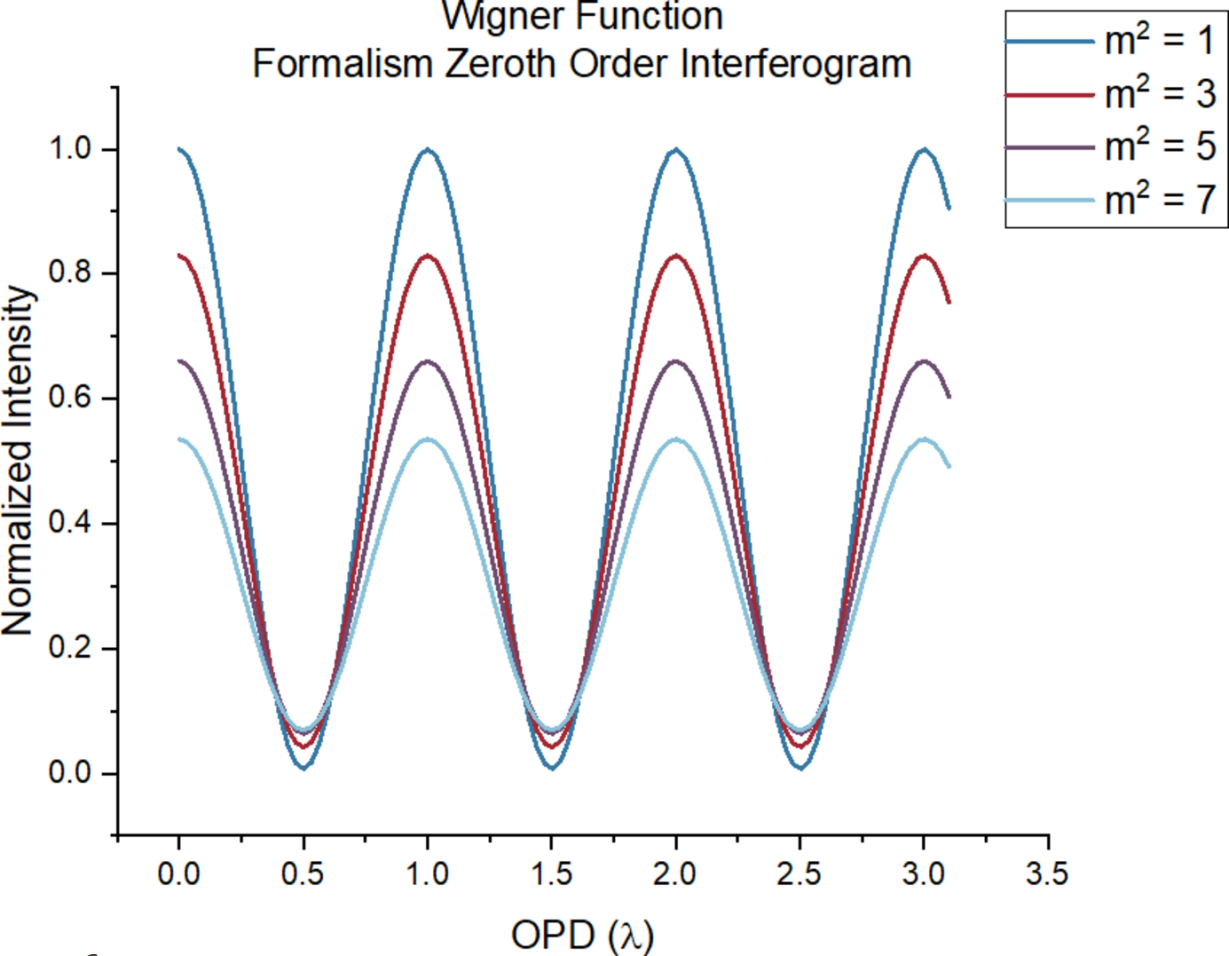
The interferogram produced by the zeroth-order peak under different *m*^2^ values. The OPD is sampled in the range from 0 to 3 times the wavelength. Simulation parameters include: *z*_1_ = 10 cm, *z*_2_ = 30 cm, *z*_4_ = 0.5 m, σ_*x*_ = 43 µm, λ = 15 nm, *a* = 5 µm, *N* = 5.

Additional interferograms for other values of *m*^2^ are shown in Fig. 6 ▸. The graph supports Yin *et al.* (2000 ▸) in stating that the interferometer operates as a wavefront-dividing system, as shown by the sensitivity of the interferograms to the coherence of the incident light field; such dependence comes from the transmission grating-like beam splitters. However, a deeper analysis of the corresponding coherent length for the corresponding values of *m*^2^ demonstrates a less stringent requirement for the coherent qualities of the incident light source. Table 2 ▸ shows the corresponding coherence width for each value of *m*^2^ in Fig. 6 ▸, calculated from equation (43).

The given slit width of 5 µm corresponds to a grating period of 10 µm. Consequently, the visible modulation in Fig. 6 ▸ indicates that the transverse coherence length of the light field does not necessarily need to exceed twice the grating period strictly, as mentioned by Yin *et al.* (2000 ▸). In other words, the coherence requirement for the incident light field can be relaxed. This means that for an incoherent or partially coherent source we can accept a higher fraction of the light than otherwise would be possible. This is already hinted at by the functional form of the complex degree of coherence for Gaussian radiation [see equation (42)]. The Gaussian functional form, rather than a Dirac delta, implies an aforementioned coherence condition that is less stringent than predicted with a classical VCZT approach.

**Figure 7:**
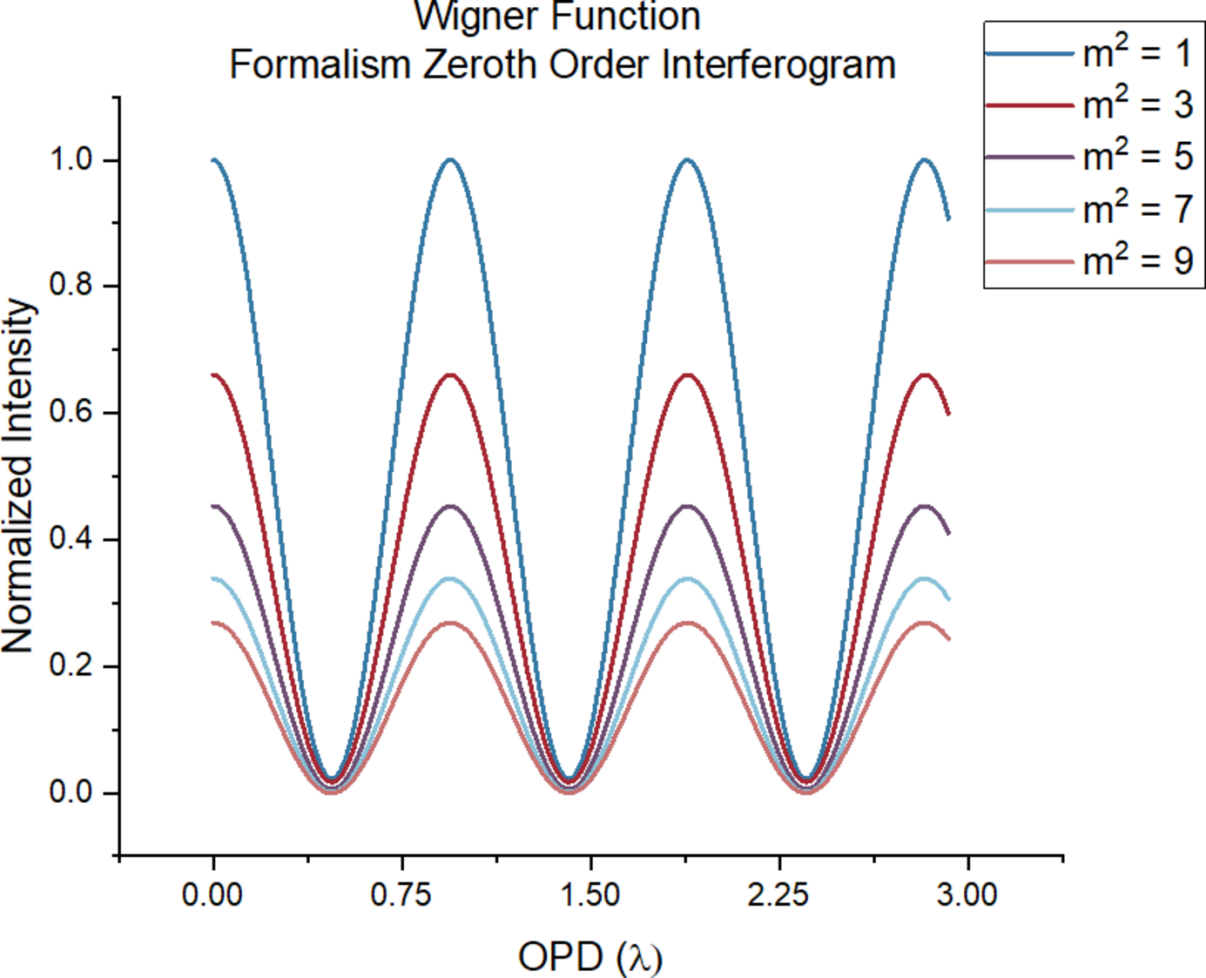
Interferogram with various values of *m*^2^. All interferograms are normalized to the *m*^2^ = 1 case to demonstrate the effect of deviation from coherence. Fixed parameters include: λ = 4.3 nm, *N* = 7, *a* = 5 µm, σ_*x*_ = 20 µm. For parameters of *z*_1_, see Table 3 ▸.

To further support this study, a corresponding simulation is performed, illustrated in Fig. 6 ▸ at a wavelength of λ = 4.3 nm, corresponding to the carbon *K*-edge. To enhance the realism of the simulation, the initial propagation distance (*z*_1_) between the source and *BS*_1_ is selected such that the angular aperture matches the FWHM of the source radiation. In other words, 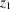 = 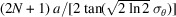 where the definition of these parameters is given in Table 1 ▸. Fig. 7 ▸ presents the resulting interferogram as a function of *m*^2^ at this wavelength.

Note that fixed parameters include: λ = 4.3 nm, *N* = 7, *a* = 5 µm, σ_*x*_ = 20 µm. Table 3 ▸ outlines the altered parameters.

**Figure 8:**
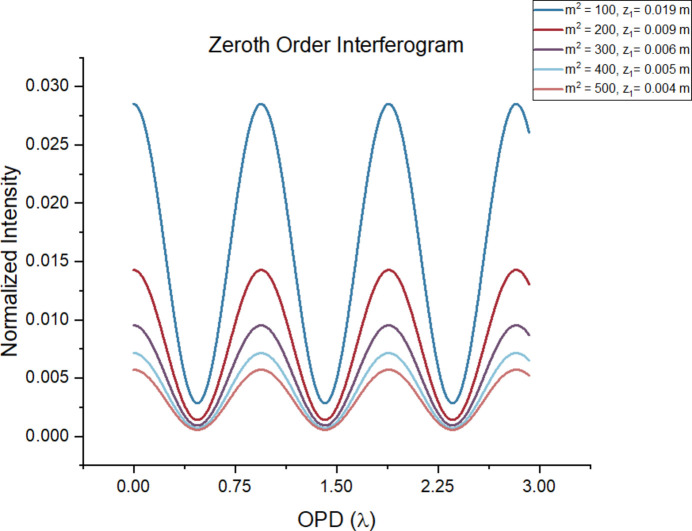
The same parameters as those presented in Fig. 7 ▸ are used with *m*^2^ in this case equal 100, 200, 300, 400 and 500. In the legend, the corresponding *z*_1_ in units of meters (calculated from matching the angular aperture with the FWHM of the incident radiation) is also presented.

Fig. 7 ▸ shows the modulation as a function of OPD for the carbon *K*-edge, for *N* = 7, *a* = 5 µm, *etc*, for *m*^2^ in the range 1 to 9. In this regime, modulation decreases as the beam becomes more incoherent, with a quasi-quadratic relationship between modulation depth and 1/*m*^2^. However, the angular aperture for *m*^2^ = 9 is only 0.15 mrad. For an aperture that is competitive with a conventional RIXS spectrograph, we would need to go to an *m*^2^ of 300, corresponding to an angular aperture of 11.4 mrad, but here the modulation as shown in Fig. 8 ▸ is only 0.01. This means that, for every one photon detected, 99 photons are diffracted into other orders. The modulation amplitude is analogous to the diffraction efficiency of a grating; however, in the case of a grating, the efficiency is independent of the aperture and can be as high as 40%. Even so, there may be some regimes where the FTS approach outperforms the traditional grating approach, such as in the ultra-high-energy resolution domain where, in principle, the maximum resolving power only depends on the maximum path length difference.

## 6. Future scope

In summary, partially coherent Gaussian radiation was propagated using the WDF formalism with the predicted interference pattern compared with the results of Yin *et al.* (2000 ▸) in the limit of *m*^2^ = 1. By analyzing peak features and comparing the interferograms predicted by these interference patterns, we have demonstrated that the WDF formalism aligns with the results in Yin *et al.* within the diffraction limit. In the process, a generalized aperture Wigner function was derived for both even and odd slits. The subsequent analysis of the interferogram reveals a less stringent requirement on the transverse coherence length of the incident light field.

An important motivation behind establishing a framework for the analysis of an FTS system is its potential advantage over grating-based spectrographs. These are intrinsically limited in terms of throughput, determined by the diffraction efficiency and aperture, and, in principle, FTS can also achieve a higher resolution. The resolving power is fundamentally limited only by the path length delay and wavelength. For example, at a 2 nm wavelength, a 2 mm path length change allows for a resolving power of 1 × 10^6^. The question is what degree of coherence is necessary in a practical geometry and what modulation would be achieved for a competitive aperture. The framework we have established can now be used to answer these fundamental questions.

Finally, we emphasize that, in this paper, we have not attempted a comprehensive evaluation of the practicality of the FTS approach for RIXS, which includes a detailed comparison with conventional grating spectrometers. A rigorous study would require accounting for photon statistics, the effect of out-of-band radiation contributing to the background in FTS, scanning speed limitations in resolving the fringes, the low scattering cross section of RIXS, and experimental issues such as synchronization. We believe that the theoretical framework established in this paper provides a solid foundation for future studies to build upon.

## 7. Related literature

The following references, not cited in the main body of the paper, have been cited in the supporting information: Cerbino (2007 ▸); Gradoni *et al.* (2014 ▸).

## Supplementary Material

Supporting Sections S1 and S2. DOI: 10.1107/S1600577525011452/mo5310sup1.pdf

## Figures and Tables

**Table 1:** Physical definitions of relevant parameters for simulation

Parameter	Definition
λ	Wavelength
*z*_1_	Distance between source and *BS*_1_
*z*_2_	Length of the *M*_1_*M*_2_ arm
*z*_4_	Distance between *BS*_2_ and observation plane
σ_*x*_	Source r.m.s. size
σ_θ_	Source r.m.s. divergence
*m* ^2^	Beam quality factor
*a*	Width of both opening and reflective bars
*N*	Number of slits/number of periods

**Table 2:** Values of the coherence width for *m*^2^ parameters in Fig. 6 ▸

*m* ^2^	*l*_*c*_ (µm)
1	∞
3	30.41
5	17.55
7	12.41

**Table 3:** Definition of source r.m.s. divergence σ_θ_ using the diffraction limit formula and *z*_1_

Source *m*^2^	σ_θ_ (µrad)	*z*_1_ (m)
1	17	1.86
3	51	0.62
5	85	0.37
7	119	0.27
9	154	0.21
