# Supplementary material for: Theoretical framework for soft X-ray Fourier transform spectroscopy using the Wigner function
Source: J Synchrotron Radiat. 2026 Jan 30;33(Pt 2):362–73. doi: 10.1107/S1600577525011452 (PMC12948022; doi:10.1107/S1600577525011452)
Supplement: Supplementary file 1 [file s-33-00362-sup1.pdf]

JOURNAL OF SYNCHROTRON RADIATION

Volume 33 (2026)

Supporting information for article:

Theoretical framework for soft X-ray Fourier transform spectroscopy using the Wigner function

Chuzida Chen, Andrew Lindburg, Honghe Ding, Antoine Wojdyla, Howard Padmore, Per-Anders Glans and Jinghua Guo

S1. Wigner Function's Connection to the VCZT and the GVCZT

Considering the definition of the spatial coherence function,

$$\Gamma(\mathbf{r_1}, \mathbf{r_2}) = \langle E(\mathbf{r_1}) E^*(\mathbf{r_2}) \rangle \tag{S1}$$

the Wigner function of a 2D, quasi-monochromatic source with average wavelength $\overline{\lambda}$ can be written as:

$$W(\mathbf{r}, \boldsymbol{\theta}) = \frac{1}{\overline{\lambda}^2} \int_{-\infty}^{\infty} \Gamma(\boldsymbol{r} - \frac{\boldsymbol{\phi}}{2}, \boldsymbol{r} + \frac{\boldsymbol{\phi}}{2}) e^{-i \frac{2\pi}{\overline{\lambda}} \boldsymbol{\phi} \cdot \boldsymbol{\theta}} d\boldsymbol{\phi} \tag{S2}$$

In turn, the spatial coherence function becomes the following (Cerbino, 2007),

$$\Gamma(\boldsymbol{r}, \boldsymbol{\phi}) = \Gamma(\boldsymbol{r} - \frac{\boldsymbol{\phi}}{2}, \boldsymbol{r} + \frac{\boldsymbol{\phi}}{2}) = \int W(\mathbf{r}, \boldsymbol{\theta}) e^{i \frac{2\pi}{\overline{\lambda}} \boldsymbol{\phi} \cdot \boldsymbol{\theta}} d\theta \tag{S3}$$

Recall that $\boldsymbol{r} = (x, y)$, $\boldsymbol{\theta} = (\theta_x, \theta_y)$ are both 2 dimensional vectors. The dummy variable $\boldsymbol{\phi}$ is also a 2D vector with the dimension of length. From here on, the vector notation will be suppressed for simplicity.

Given the propagation law of the Wigner function, we can derive a similar rule that governs the evolution of the spatial coherence function. Substituting the propagation law of WDF into the definition above, we have

$$\begin{aligned} \Gamma(r, \phi; z) &= \int W(r, \theta; z) e^{i \frac{2\pi}{\overline{\lambda}} \phi \theta} d\theta \\ &= \int W(r - z\theta, \theta; 0) e^{i \frac{2\pi}{\overline{\lambda}} \phi \theta} d\theta \end{aligned} \tag{S4}$$

Equation (S2) tells us that

$$W(r - z\theta, \theta, 0) = \frac{1}{\overline{\lambda}^2} \int \Gamma(r - z\theta, \phi'; 0) e^{-i \frac{2\pi}{\overline{\lambda}} \phi' \theta} d\phi' \tag{S5}$$

Substitute this into expression (S4),

$$\Gamma(r, \phi; z) = \frac{1}{\overline{\lambda}^2} \int \int \Gamma(r - z\theta, \phi'; 0) e^{-i \frac{2\pi}{\overline{\lambda}} \phi' \theta} e^{i \frac{2\pi}{\overline{\lambda}} \phi \theta} d\phi' d\theta \tag{S6}$$

This is the general propagation law of the spatial coherence function predicted by the Wigner function, demonstrated in Gradoni *e*t al. (2014). The next step is to introduce approximations that relate the spatial coherence function to the source intensity distribution.

The spatial coherence function of an incoherent light field is approximately (Cerbino, 2007)

$$\Gamma(\boldsymbol{r_1}, \boldsymbol{r_2}) = \kappa I(\overline{\boldsymbol{r}}) \delta(\Delta \boldsymbol{r}) \tag{S7}$$

Assuming $\boldsymbol{r_1}$ and $\boldsymbol{r_2}$ are on the same z-plane, $\overline{\boldsymbol{r}} = \frac{\boldsymbol{r_1} + \boldsymbol{r_2}}{2}$ and $\Delta \boldsymbol{r} = \boldsymbol{r_2} - \boldsymbol{r_1}$. The constant κ

is introduced to ensure that the volume of the approximated function is the same as its original form, which involves first-kind Bessel functions of the first order (Goodman, 2000). Along with the identity,

$$\begin{aligned}\Gamma(\overline{\boldsymbol{r}}, \Delta\boldsymbol{r}) &= \Gamma(\overline{\boldsymbol{r}} - \frac{\Delta\boldsymbol{r}}{2}, \overline{\boldsymbol{r}} + \frac{\Delta\boldsymbol{r}}{2}) \\ &= \Gamma(\boldsymbol{r}_1, \boldsymbol{r}_2)\end{aligned}$$

the integrand within equation (S6) can be written as

$$\Gamma(r - z\theta, \phi'; 0) = \kappa I(r - z\theta)\delta(\phi') \tag{S8}$$

Then, equation (S6) can be simplified as follows:

$$\begin{aligned}\Gamma(r, \phi; z) &= \frac{\kappa}{\overline{\lambda}^2} \int\int I(r - z\theta)\delta(\phi')e^{-i\frac{2\pi}{\overline{\lambda}}\phi'\theta}e^{i\frac{2\pi}{\overline{\lambda}}\phi\theta}d\phi' d\theta \\ &= \frac{\kappa}{\overline{\lambda}^2}\int I(r - z\theta)e^{i\frac{2\pi}{\overline{\lambda}}\phi\theta}d\theta\end{aligned} \tag{S9}$$

We then make the substitution of $r - z\theta = S$. Recall that r and θ are vectors, which implies the vectorial nature of S. Under this substitution, $z^2 d\theta = dS$; variables in the x and y direction would each contribute a factor of $-z$, hence, when multiplied, we have z^2. Bringing back the vector notations, we finally arrive at

$$\Gamma(\boldsymbol{r}, \boldsymbol{\phi}; z) = \frac{\kappa}{(\overline{\lambda}z)^2}\int I(\boldsymbol{S})e^{i\frac{2\pi}{\overline{\lambda}}\boldsymbol{\phi}\cdot\frac{\boldsymbol{r}-\boldsymbol{S}}{z}}d\boldsymbol{S} \tag{S10}$$

We can then identify that $\boldsymbol{\phi}$ is the negative of a two-dimensional displacement $\Delta\boldsymbol{r} = (\Delta x, \Delta y) = \boldsymbol{r}_2 - \boldsymbol{r}_1$; the position vector r can be substituted with the average position vector $\overline{\boldsymbol{r}} = (\overline{x}, \overline{y}) = \frac{\boldsymbol{r}_1 + \boldsymbol{r}_2}{2}$. The expression then becomes

$$\Gamma(\overline{\boldsymbol{r}}, \Delta\boldsymbol{r}; z) = \frac{\kappa e^{-i\frac{2\pi}{\overline{\lambda}z}\overline{\boldsymbol{r}}\cdot\Delta\boldsymbol{r}}}{(\overline{\lambda}z)^2}\int I(\boldsymbol{S})e^{i\frac{2\pi}{\overline{\lambda}z}\Delta\boldsymbol{r}\cdot\boldsymbol{S}}d\boldsymbol{S} \tag{S11}$$

Noting the relation that

$$\begin{aligned}\psi &= \frac{\pi}{\overline{\lambda}z}[(x_2^2 + y_2^2) - (x_1^2 + y_1^2)] \\ &= \frac{\pi}{\overline{\lambda}z}[(x_2^2 - x_1^2) + (y_2^2 - y_1^2)] \\ &= \frac{\pi}{\overline{\lambda}z}[(x_1 + x_2)(x_2 - x_1) + (y_1 + y_2)(y_2 - y_1)] \\ &= \frac{2\pi}{\overline{\lambda}z}(\overline{x}\Delta x + \overline{y}\Delta y) \\ &= \frac{2\pi}{\overline{\lambda}z}\overline{\boldsymbol{r}}\cdot\Delta\boldsymbol{r}\end{aligned}$$

equation (S11) is the exact form of VCZT defined in Goodman (2000).

The GVCZT models the propagation of spatial coherence functions associated with a specific class of partially coherent radiation, having the spatial coherence functions of the form:

$$\Gamma(\boldsymbol{r_1}, \boldsymbol{r_2}) = I(\overline{\boldsymbol{r}})\mu(\Delta \boldsymbol{r}) \tag{S12}$$

In the expression above, the function μ is called the complex coherence factor. This function is essentially the spatial coherence function normalized by the respective intensities of the field at the two positions (Goodman, 2000). μ has the property that

$$0 \leq |\mu| \leq 1$$

Sources associated with the spatial coherence function above are classified as planar quasi-homogeneous. As it turns out, the substitutions that lead to equation (S11) also appropriately describe these quasi-homogeneous sources (Cerbino, 2007). Again suppressing vectorial notation, insertion of equation (S12) into equation (S6) leads to

$$\Gamma_p(\overline{r}, \Delta r; z) = -\frac{1}{\overline{\lambda}^2} \int \int I(\overline{r} - z\theta)\mu(-\Delta r') e^{i\frac{2\pi}{\overline{\lambda}}\theta(\Delta r' - \Delta r)} d\Delta r' d\theta \tag{S13}$$

The subscript p indicates that this is the spatial coherence function of a partially coherent source. Making the same substitution of $S = \overline{r} - z\theta$, we find the following.

$$= -\frac{1}{(\overline{\lambda}z)^2} \int \int I(S)\mu(-\Delta r') e^{i\frac{2\pi}{\overline{\lambda}z}(\overline{r} - S)(\Delta r' - \Delta r)} d\Delta r' dS$$

One can then switch the order of integration, simplify the exponent, and yield the following:

$$\begin{aligned} &= -\frac{1}{(\overline{\lambda}z)^2} \int \int I(S)\mu(-\Delta r') e^{i\frac{2\pi}{\overline{\lambda}z}S(\Delta r - \Delta r')} \\ &\cdot e^{-i\frac{2\pi}{\overline{\lambda}z}\overline{r}\Delta r} e^{i\frac{2\pi}{\overline{\lambda}z}\overline{r}\Delta r'} dS d\Delta r' \\ &= -\int \mu(-\Delta r') e^{i\frac{2\pi}{\overline{\lambda}z}\overline{r}\Delta r'} \frac{e^{-i\frac{2\pi}{\overline{\lambda}z}\overline{r}\Delta r}}{(\overline{\lambda}z)^2} \\ &\cdot \int I(S) e^{i\frac{2\pi}{\overline{\lambda}z}S\Delta r} e^{-i\frac{2\pi}{\overline{\lambda}z}S\Delta r'} dS d\Delta r' \end{aligned}$$

The second part of the above expression is precisely the VCZT integral multiplied by an exponential. Thus, using the Fourier shift property:

$$= -\int \mu(-\Delta r') e^{i\frac{2\pi}{\overline{\lambda}z}\overline{r}\Delta r'} \Gamma_{ic}(\overline{r}, \Delta r + \Delta r') d\Delta r'$$

Goodman's derivation of the GVCZT also assumes the condition that $z >> \frac{Dd_c}{\lambda}$, with z being the

screen-detector separation, D is the maximum dimension of the source, d_c is the dimension of the coherence area, and λ is the wavelength. This criterion implies that $\frac{\overline{r}\Delta r'}{\overline{\lambda} z} \approx 0$ (Goodman, 2000). Therefore, making the substitution $-\Delta r' \longrightarrow \rho$, we arrive at the final expression:

$$\Gamma_p(\overline{r}, \Delta r; z) = \int \mu(\rho)\Gamma_{ic}(\overline{r}, \Delta r - \rho) d\rho \tag{S14}$$

This is the convolution form of the GVCZT shown by Cerbino (2007). This implies, physically, that the propagated mutual coherence function of a partially coherent radiation is the convolution product between its complex coherence factor and the propagated mutual coherence function of an incoherent source of the same parameters.

S2 Mathematics for Propagating the Gaussian Wigner Function

Even N Slits

Given the source Wigner function as stated in equation (33), at BS1, the WDF would have become:

$$
\begin{aligned}
W_0(x,\theta;z_1) &= W_0(x-z_1\theta,\theta;0) \\
&= \frac{1}{2\pi\sigma_x\sigma_\theta}e^{-\frac{(x-z_1\theta)^2}{2\sigma_x^2}-\frac{\theta^2}{2\sigma_\theta^2}} \\
&= \frac{1}{2\pi\sigma_x\sigma_\theta}H_1(x)e^{\frac{(\theta-\psi_1)^2}{2\sigma_{\theta1}^{\prime 2}}}
\end{aligned}
\tag{S15}
$$

parameters $\sigma_{\theta1}^{\prime 2}$ and functions $\psi_1(x)$, $H_1(x)$ are defined according to equation (35) by substituting z with z_1, which is the drift distance between the source and the first aperture. According to our calculation steps, the Wigner function associated with the lights at both arms of the interferometer will be half of the incident Wigner function $W(x,\theta;z_1)$.

From this, the two Wigner functions propagate a distance of z_2 and z_3, respectively. Following the free space propagation rule, the two Wigner functions incident at BS_2 would be:

$$
\begin{aligned}
W_1(x,\theta;z_2) &= W_1(x-z_2\theta,\theta;0) \\
&= \frac{1}{2}W_0(x-(z_1+z_2)\theta,\theta;0)
\end{aligned}
$$

$$
\begin{aligned}
W_2(x,\theta;z_3) &= W_2(x-z_3\theta,\theta;0) \\
&= \frac{1}{2}W_0(x-(z_1+z_3)\theta,\theta;0)
\end{aligned}
$$

Therefore, exploiting equation (36), BS_2 modifies the beam of the M_1M_2 arm into the following form:

$$W_1'(x,\theta;0) = \frac{1}{4} W_0(x-(z_1+z_2)\theta,\theta;0) \cdot \sum_{m=0}^{\frac{N-2}{2}} \sum_{n=0}^{\frac{N-2}{2}}$$

$$\begin{cases}
[\mathrm{Im}\{ierf(\frac{\sigma'_{\theta,2}(\frac{2\pi}{\lambda}[-2||x|+\frac{(n+m+1)b}{2}|+a+(n-m)b])+i(\hat{\theta}_2-\hat{\psi}_2)}{\sqrt{2}})\} \\
+\mathrm{Im}\{ierf(\frac{\sigma'_{\theta,2}(\frac{2\pi}{\lambda}[-2||x|+\frac{(n+m+1)b}{2}|+a-(n-m)b])+i(\hat{\theta}_2-\hat{\psi}_2)}{\sqrt{2}})\}] \\
\;,\; ||x|+\frac{(n+m+1)b}{2}|<\frac{a}{2} \\
\\
[\mathrm{Im}\{ierf(\frac{\sigma'_{\theta,2}(\frac{2\pi}{\lambda}[-2||x|+\frac{(m-n)b}{2}|+a+(n+m+1)b])+i(\hat{\theta}_2-\hat{\psi}_2)}{\sqrt{2}})\} \\
+\mathrm{Im}\{ierf(\frac{\sigma'_{\theta,2}(\frac{2\pi}{\lambda}[-2||x|+\frac{(m-n)b}{2}|+a-(n+m+1)b])+i(\hat{\theta}_2-\hat{\psi}_2)}{\sqrt{2}})\}] \\
\;,\; ||x|+\frac{(m-n)b}{2}|<\frac{a}{2} \\
\\
[\mathrm{Im}\{ierf(\frac{\sigma'_{\theta,2}(\frac{2\pi}{\lambda}[-2||x|+\frac{(n-m)b}{2}|+a+(n+m+1)b])+i(\hat{\theta}_2-\hat{\psi}_2)}{\sqrt{2}})\} \\
+\mathrm{Im}\{ierf(\frac{\sigma'_{\theta,2}(\frac{2\pi}{\lambda}[-2||x|+\frac{(n-m)b}{2}|+a-(n+m+1)b])+i(\hat{\theta}_2-\hat{\psi}_2)}{\sqrt{2}})\}] \\
\;,\; ||x|+\frac{(n-m)b}{2}|<\frac{a}{2} \\
\\
[\mathrm{Im}\{ierf(\frac{\sigma'_{\theta,2}(\frac{2\pi}{\lambda}[-2||x|+\frac{-(n+m+1)b}{2}|+a+(m-n)b])+i(\hat{\theta}_2-\hat{\psi}_2)}{\sqrt{2}})\}+ \\
\mathrm{Im}\{ierf(\frac{\sigma'_{\theta,2}(\frac{2\pi}{\lambda}[-2||x|+\frac{-(n+m+1)b}{2}|+a-(m-n)b])+i(\hat{\theta}_2-\hat{\psi}_2)}{\sqrt{2}})\}] \\
\;,\; ||x|+\frac{-(n+m+1)b}{2}|<\frac{a}{2}
\end{cases}$$

$$\sigma'^2_{\theta,2} \equiv \frac{\sigma_x^2\sigma_\theta^2}{((z_1+z_2)^2\sigma_\theta^2+\sigma_x^2)}$$

$$\psi_2 = \frac{(z_1+z_2)x\sigma_\theta^2}{((z_1+z_2)^2\sigma_\theta^2+\sigma_x^2)}$$

$$\hat{\psi}_2 = \psi_2/\sigma'_{\theta,2}\;,\; \hat{\theta}_2 = \theta/\sigma'_{\theta,2} \tag{S16}$$

As for the light field of the M_3M_4 arm, BS_2 modifies the light field according to equation (31). In this case, the Wigner function corresponding to the second arm after BS_2 is given by the following expression.

$$W_2'(x,\theta;0) = \frac{1}{4} W_0(x-(z_1+z_3)\theta,\theta;0) \cdot \sum_{m=0}^{\frac{N-2}{2}} \sum_{n=0}^{\frac{N-2}{2}}$$

$$\begin{cases}
[\mathrm{Im}\{ierf(\frac{\sigma'_{\theta,2}(\frac{2\pi}{\lambda}[-2||x-a|+\frac{(n+m+1)b}{2}|+a+(n-m)b])+i(\hat{\theta}_2-\hat{\psi}_2)}{\sqrt{2}})\} \\
+\mathrm{Im}\{ierf(\frac{\sigma'_{\theta,2}(\frac{2\pi}{\lambda}[-2||x-a|+\frac{(n+m+1)b}{2}|+a-(n-m)b])+i(\hat{\theta}_2-\hat{\psi}_2)}{\sqrt{2}})\}] \\
\;,\; ||x-a|+\frac{(n+m+1)b}{2}|<\frac{a}{2} \\
\\
[\mathrm{Im}\{ierf(\frac{\sigma'_{\theta,2}(\frac{2\pi}{\lambda}[-2||x-a|+\frac{(m-n)b}{2}|+a+(n+m+1)b])+i(\hat{\theta}_2-\hat{\psi}_2)}{\sqrt{2}})\} \\
+\mathrm{Im}\{ierf(\frac{\sigma'_{\theta,2}(\frac{2\pi}{\lambda}[-2||x-a|+\frac{(m-n)b}{2}|+a-(n+m+1)b])+i(\hat{\theta}_2-\hat{\psi}_2)}{\sqrt{2}})\}] \\
\;,\; ||x-a|+\frac{(m-n)b}{2}|<\frac{a}{2} \\
\\
[\mathrm{Im}\{ierf(\frac{\sigma'_{\theta,2}(\frac{2\pi}{\lambda}[-2||x-a|+\frac{(n-m)b}{2}|+a+(n+m+1)b])+i(\hat{\theta}_2-\hat{\psi}_2)}{\sqrt{2}})\} \\
+\mathrm{Im}\{ierf(\frac{\sigma'_{\theta,2}(\frac{2\pi}{\lambda}[-2||x-a|+\frac{(n-m)b}{2}|+a-(n+m+1)b])+i(\hat{\theta}_2-\hat{\psi}_2)}{\sqrt{2}})\}] \\
\;,\; ||x-a|+\frac{(n-m)b}{2}|<\frac{a}{2} \\
\\
[\mathrm{Im}\{ierf(\frac{\sigma'_{\theta,2}(\frac{2\pi}{\lambda}[-2||x-a|+\frac{-(n+m+1)b}{2}|+a+(m-n)b])+i(\hat{\theta}_2-\hat{\psi}_2)}{\sqrt{2}})\}+ \\
\mathrm{Im}\{ierf(\frac{\sigma'_{\theta,2}(\frac{2\pi}{\lambda}[-2||x-a|+\frac{-(n+m+1)b}{2}|+a-(m-n)b])+i(\hat{\theta}_2-\hat{\psi}_2)}{\sqrt{2}})\}] \\
\;,\; ||x-a|+\frac{-(n+m+1)b}{2}|<\frac{a}{2}
\end{cases}$$

$$\sigma'^2_{\theta,2} \equiv \frac{\sigma_x^2\sigma_\theta^2}{((z_1+z_3)^2\sigma_\theta^2+\sigma_x^2)}$$

$$\psi_2 = \frac{(z_1+z_3)x\sigma_\theta^2}{((z_1+z_3)^2\sigma_\theta^2+\sigma_x^2)}$$

$$\hat{\psi}_2 = \psi_2/\sigma'_{\theta,2}\;,\; \hat{\theta}_2 = \theta/\sigma'_{\theta,2} \tag{S17}$$

As discussed previously, the combined Wigner function will be equation (32) with its $W_1'(x,\theta;0)$ being equation (S16) and $W_2'(x,\theta;0)$ being equation (S17).

Odd N Slits

Following the same procedure, we change the form of equation (27) into the following form:

$$W_{t,N}(x,\theta) = \sum_{m=-\frac{N-1}{2}}^{\frac{N-1}{2}} \sum_{n=-\frac{N-1}{2}}^{\frac{N-1}{2}} \frac{1}{2\pi\theta} \sin(\frac{2\pi}{\lambda}\theta[-2||x| + \frac{(-n-m)b}{2}| + a + (m-n)b])$$
$$+ \sum_{m=-\frac{N-1}{2}}^{\frac{N-1}{2}} \sum_{n=-\frac{N-1}{2}}^{\frac{N-1}{2}} \frac{1}{2\pi\theta} \sin(\frac{2\pi}{\lambda}\theta[-2||x| + \frac{(-n-m)b}{2}| + a - (m-n)b]),$$
$$||x| + \frac{(-n-m)b}{2}| < \frac{a}{2}$$

In this case, W_t, a would simply be:

$$W_{t,a}(x,\theta) = \sum_{m=-\frac{N-1}{2}}^{\frac{N-1}{2}} \sum_{n=-\frac{N-1}{2}}^{\frac{N-1}{2}} \frac{1}{2\pi\theta} \sin(\frac{2\pi}{\lambda}\theta[-2||x-a| + \frac{(-n-m)b}{2}| + a + (m-n)b])$$
$$+ \sum_{m=-\frac{N-1}{2}}^{\frac{N-1}{2}} \sum_{n=-\frac{N-1}{2}}^{\frac{N-1}{2}} \frac{1}{2\pi\theta} \sin(\frac{2\pi}{\lambda}\theta[-2||x-a| + \frac{(-n-m)b}{2}| + a - (m-n)b]),$$
$$||x-a| + \frac{(-n-m)b}{2}| < \frac{a}{2}$$

Performing the convolution over the angular variable and simplifying yields the Wigner function associated with the M_1M_2 arm after BS_2:

$$
\begin{aligned}
W_1'(x,\theta;0) =& \frac{1}{4}W_0(x-(z_1+z_2)\theta,\theta;0)\cdot[\sum_{m=-\frac{N-1}{2}}^{\frac{N-1}{2}}\sum_{n=-\frac{N-1}{2}}^{\frac{N-1}{2}} \\
&\mathrm{Im}\{ierf(\frac{\sigma'_{\theta 2}\frac{2\pi}{\lambda}[-2||x|+\frac{(-n-m)b}{2}|+a+(m-n)b]-i\frac{\theta-\psi_1}{\sigma'_{\theta 2}}}{\sqrt{2}})\} \\
&+\mathrm{Im}\{ierf(\frac{\sigma'_{\theta 2}\frac{2\pi}{\lambda}[-2||x|+\frac{(-n-m)b}{2}|+a-(m-n)b]-i\frac{\theta-\psi_1}{\sigma'_{\theta 2}}}{\sqrt{2}})\}], \qquad \text{(S18)} \\
&||x|+\frac{(-n-m)b}{2}|<\frac{a}{2}
\end{aligned}
$$

$$
\psi_2=\frac{(z_1+z_2)x\sigma_\theta^2}{(\sigma_x^2+(z_1+z_2)^2\sigma_\theta^2)}, \qquad \sigma_{\theta 2}'^2=\frac{\sigma_x^2\sigma_\theta^2}{(\sigma_x^2+(z_1+z_2)^2\sigma_\theta^2)}
$$

Similarly, the aperture-modified Wigner function associated with the M_3M_4 arm after encountering BS_2 would be the following:

$$W_2'(x,\theta;0) = \frac{1}{4}W_0(x-(z_1+z_3)\theta,\theta;0)\cdot[\sum_{m=-\frac{N-1}{2}}^{\frac{N-1}{2}}\sum_{n=-\frac{N-1}{2}}^{\frac{N-1}{2}} \mathrm{Im}\{ierf(\frac{\sigma'_{\theta 2}\frac{2\pi}{\lambda}[-2||x-a|+\frac{(-n-m)b}{2}|+a+(m-n)b]-i\frac{\theta-\psi_1}{\sigma'_{\theta 2}}}{\sqrt{2}})\} + \mathrm{Im}\{ierf(\frac{\sigma'_{\theta 2}\frac{2\pi}{\lambda}[-2||x-a|+\frac{(-n-m)b}{2}|+a-(m-n)b]-i\frac{\theta-\psi_1}{\sigma'_{\theta 2}}}{\sqrt{2}})\}], \quad \text{(S19)}$$

$$||x-a|+\frac{(-n-m)b}{2}|<\frac{a}{2}$$

$$\psi_2 = \frac{(z_1+z_3)x\sigma_\theta^2}{(\sigma_x^2+(z_1+z_3)^2\sigma_\theta^2)}, \qquad \sigma_{\theta 2}'^2 = \frac{\sigma_x^2\sigma_\theta^2}{(\sigma_x^2+(z_1+z_3)^2\sigma_\theta^2)}$$

The final combined Wigner function after BS_2 still follows equation (32), defined by the two above equations.

References

Cerbino, R. (2007). *Phys. Rev. A*, **75**, 053815.

Goodman, J. W. (2000). *Statistical Optics.* John Wiley Sons.

Gradoni, G., Creagh, S. C. Tanner, G. (2014). *Proceedings of the 2014 IEEE International Symposium on Electromagnetic Compatibility (EMC2014)*, 4-8 August 2014, Raleigh, NC, USA, pp. 882-887.
